# Embracing the future: The evolution of systematic reviews and meta-analyses in periodontology

**DOI:** 10.34172/japid.2023.025

**Published:** 2023-12-18

**Authors:** Fatemeh Pournaghi Azar, Morteza Ghojazadeh

**Affiliations:** ^1^Research Center for Evidence-Based Medicine, Tabriz University of Medical Sciences, Tabriz, Iran; ^2^Neurosciences Research Center (NSRC), Tabriz University of Medical Sciences, Tabriz, Iran

## Dear Editor;

 With the growing emphasis on evidence-based practice, systematic reviews and meta-analyses have become crucial tools for synthesizing research findings to guide clinical decision-making. Due to significant advancements in dentistry in recent years, as we look toward the future, these articles will continue to play an essential role in shaping this advancing field, and the significance of systematic reviews and meta-analyses cannot be underestimated.^1^

 These studies are critical in providing a comprehensive overview of a particular topic. Systematic reviews and meta-analyses serve as powerful tools for assessing the effectiveness of numerous treatments, diagnostic techniques, and preventive measures, leading to evidence-based decision-making in clinical oral health practice and hold promises for further modifications and improvements.

 Also, technological advancements are expected to revolutionize the process of systematic reviews and meta-analyses.^2,3^ Emerging technological trends have expedited the process of literature retrieval and data extraction. The future landscape in periodontology is shaped by advanced trends that are composed to reconsider research conduction and evidence-synthesizing methods. Using the power of artificial intelligence (AI) and computational algorithms, researchers can automate certain aspects, including screening and selecting relevant studies.^4^ Moreover, using data visualization tools and interactive platforms can improve the availability of review findings, making them more user-friendly for both researchers and clinicians. Therefore, AI can expedite the overall process and potentially uncover new patterns and associations within the oral health and periodontal literature. On the other hand, the arrival of the big data era has opened up new avenues for conducting comprehensive and robust systematic reviews and meta-analyses.^5^ The combination of real-world evidence and big data sources enriches the depth of evidence synthesis in periodontology. With the proliferation of electronic health records, patient registries, and population-based databases, several data sources can be reached to perform systematic reviews and meta-analyses, resulting in more comprehensive outcomes in dental practice.^4,5^

 There are multifaceted advantages; firstly, the comprehensive and systematic approach for synthesizing evidence provides a more comprehensive understanding of the efficacy and safety of diverse periodontal interventions. Combining data from multiple studies enables researchers and practitioners to recognize trends, patterns, and discrepancies.^6^ Moreover, these methods minimize bias risk and, therefore, increase the statistical power of the findings, which results in improved reliability of the drawn evidence. Furthermore, these studies can guide researchers in future studies by identifying research gaps and prioritizing investigation areas in periodontology.^1,7^

 However, certain challenges need to be addressed. Keeping reviews up to date despite the rapid pace of research and the sheer volume of publications can be one of the primary obstacles. Collaboration among researchers, clinicians, and policy-makers is a critical step in confirming the pertinency of review findings in real-world settings. In some areas of periodontal research, the lack of high-quality studies and standardized outcome measurements can pose challenges in directing reliable systematic reviews and meta-analyses. Additionally, the heterogeneity of study designs, populations, and methodologies across oral health, especially periodontal articles, makes the data synthesis process complicated. Addressing these challenges requires a multidisciplinary approach and the incorporation of various expertise.^1,3,8^

 Systematic reviews and meta-analyses provide a detailed foundation for establishing best practices and guidelines in healthcare, promising more informed and evidence-based decisions in this field with more optimized patient outcomes and safety. Furthermore, integrating systematic reviews and meta-analyses into evidence-based practice raises a culture of continuous quality improvement and professional development within the periodontal community.^9^ Critically appraising the existing evidence and determining uncertainties cater to the need for ongoing research and novelties in periodontal practice.

 Association with best practices is fundamental to ensure the reliability of systematic reviews and meta-analyses. One of the first-line necessities is a well-defined research question and clear inclusion and exclusion criteria. The reproducibility of data extraction methods, as well as comprehensive statistical analysis techniques, are crucial for maintaining the validity of systematic reviews and meta-analyses.^2,9^

 Moreover, a multidisciplinary team comprising dental professionals, methodologists, statisticians, and subject experts can perform better in conducting these studies. Lastly, peer reviews and critical appraisal of systematic reviews and meta-analyses by the periodontology research community can enhance the validity and reliability of these methodologies.

 The Future Landscape of Systematic Reviews and Meta-Analysis in Periodontology holds great promise for potential advancements in evidence-based practice, clinical decision-making, and patient care. With the convergence of technological innovations, the integration of big data, interdisciplinary association, and a commitment to evolving standards and methodologies, the periodontology community will witness more efficient, comprehensive, and clinically relevant research.

## Competing Interests

 The authors declare that they have no financial and non-financial competing interests with regard to the publication of their work.

## Funding

 Not applicable.
